# The minimum requirements for prenatal “confirmatory” diagnosis of fetal aneuploidy

**DOI:** 10.1002/pmf2.70440

**Published:** 2026-08-27

**Authors:** Jose Carlos Ferreira, Brynn Levy, Peter Benn

**Affiliations:** ^1^ Departamento de Obstetrícia e Ginecologia Faculdade de Medicina Universidade de Lisboa Lisbon Portugal; ^2^ College of Physicians and Surgeons Columbia University Medical Center & the New York Presbyterian Hospital New York New York USA; ^3^ Department of Obstetrics and Gynecology University of Connecticut Health Center Farmington Connecticut USA

A recent American college of obstetricians and gynecologists (ACOG) industry symposium, a widely circulated White Paper, and media coverage have presented cell‐based noninvasive prenatal testing using circulating extra‐villous trophoblasts (cEVTs) as a “confirmation test” that “bridges the gap between screening and invasive diagnostics” [1, 2]. In this Viewpoint, we discuss the dangers of uncritically accepting the premise that cEVT testing is sufficient for confirming a diagnosis of aneuploidy.

The presentation at the ACOG meeting covered a proof‐of‐concept study involving only 16 pregnancies where cEVTs were identified by immunofluorescence imaging followed by whole‐genome amplification to evaluate the presence or absence of chromosome abnormality. Interpretation was based on analysis of an average of three cEVTs per case [2]. The study did not address patient inclusion/exclusion criteria, the technical criteria used to establish a chromosome imbalance, or the criteria used to unambiguously distinguish between conceptus and maternal cells. On the basis of this study, the testing is reported to be commercially available as an adjunct in cases with positive cell‐free DNA results, available from May 28, 2026, and is intended for “patients who cannot, or choose not to, pursue invasive diagnostic testing.”

To understand the concerns about not providing a fully diagnostic test, some understanding of traditional prenatal cytogenetic diagnosis is necessary. From as early as the 1980s, it was recognized that confined placental mosaicism (CPM, the phenomenon in which the placenta may show cells with a chromosome complement that differs from the fetus) could occasionally confound prenatal diagnosis of fetal aneuploidy [3]. Large prospective studies of chorionic villus samples (CVS) as well as data accumulated from cytogenetic laboratories showed that CPM primarily, but not exclusively, involved the trophoblastic cell lineage. In some cases, there could be a complete discordance between the karyotype observed for trophoblasts and that for the placental mesenchyme or the fetus [4]. Although trophoblast analysis provided a quick evaluation because the cells were actively mitotic (i.e., analyzable following direct chromosome preparations or with short‐term culture), additional testing through the outgrowth of mesenchymal cells (long‐term cell culture) was required [4, 5]. Analysis of amniotic fluid cells was always viewed as the definitive prenatal diagnostic test, with a specified number of cell counts required for standard testing, and extended analyses required when there was a suspicion of mosaicism.

With the further experience gained from a large dataset of CVS tests in which suspected mosaic cases routinely received a confirmatory amniocentesis, it was possible to establish the false‐discovery rates (incorrect positive calls expressed as a percentage of all positive test calls) when cytogenetic testing was based on trophoblasts alone [6]. For trisomies 21, 18, and 13 and monosomy‐X, these false‐discovery rates were 0.4%, 2.4%, 8.1%, and 34.6%, respectively. False discoveries are therefore relatively common when analysis was based only on trophoblasts. International technical standards for CVS prenatal diagnosis of aneuploidy require the inclusion of mesenchyme cell analysis because it represents the tissue developmentally closest to the fetus.

Prenatal screening of genetic disorders has always been founded on the principles that screening tests (including cell‐free DNA and cEVT–based tests) do not unambiguously distinguish between affected and unaffected pregnancies. “Confirmatory” diagnostic tests are required for those with positive screening results. The concept of a “confirmatory” test has largely been used to describe an independent test that provides a definitive diagnosis *for the fetus*. It is therefore somewhat misleading to characterize the evaluation of cEVTs as a “confirmation test” for cell‐free DNA test‐positive pregnancies because, in many instances, it merely reaffirms the presence of abnormal trophoblasts. *Restated, a positive cEVT analysis does not “confirm” true fetal abnormality*. Moreover, a negative cEVT test would not necessarily exclude a clinically significant true fetal and placental mosaicism because the analysis of only a few trophoblasts, by chance, may be insufficient to detect the subpopulation that is aneuploid.

Following a positive cf‐DNA test for fetal aneuploidy, there is a high risk for abnormality (i.e., the cf‐DNA screening test has excellent performance with high positive predictive values), but professional guidelines still recommend a fully diagnostic test [7]. The data presented in the study are not sufficient to draw any conclusions that, for cf‐DNA test‐positive pregnancies, the risk of an affected pregnancy is materially refined by adding cEVT testing. We recognize that following a prenatal screening test, access to timely fully diagnostic testing can be a significant problem, particularly in locations that have restrictive laws for pregnancy termination or where there is limited access to advanced prenatal services. However, these difficulties are not ameliorated by offering a test, such as cEVT analysis, that does not provide a definitive diagnosis.

In other prenatal genetic testing contexts, cEVT analysis is poised to have a significant role. There is now a prospective, blinded, and published clinical trial involving over 1300 pregnancies that demonstrates cEVTs can result in effective screening, but not diagnosis, for a broad range of clinically significant microdeletions and microduplications [8]. Potential also exists for the detection of monogenic disorders. These latter genetic disorders differ from aneuploidy in their etiology, with CPM being a lesser concern. Each subcategory of genetic disease (aneuploidy, copy number variation, and monogenic disorders) is subject to distinct analytic and interpretation considerations.

The onus of determining whether a new prenatal test has clinical value cannot be placed solely on the provider. Clinicians cannot be expected to have complete breadth of knowledge covering the molecular biology, pathology of the fetoplacental unit, epidemiology, technical procedures, testing limitations, and statistical robustness. Indeed, these details are not always shared by the laboratories that provide the tests. In this context, we believe professional societies have an ethical obligation to provide timely evaluation of new tests, generate guidelines, and educate their society members on appropriate use. Inadequate industry self‐regulation, combined with inadequate professional society oversight, is likely to lead to renewed calls for government regulation.

It is critically important that test validation is based on substantive, peer‐reviewed studies with clearly specified limitations. Clinical trials need to precede the introduction of the test into clinical practice. While medical conferences serve as forums for scientific exchange, delegates must remain keenly aware of the current environment of commercial promotion of insufficiently validated tests. Given the landscape of intense commercial competition in prenatal genetic testing, clinical providers must remain mindful of the risks of misdiagnosis posed by inadequate studies and imprecise marketing claims.

## AUTHOR CONTRIBUTIONS

**Jose Carlos Ferreira**: Conceptualization; writing—original draft; writing—review and editing. **Brynn Levy**: Conceptualization; writing–original draft; writing—review and editing. **Peter Benn**: Conceptualization; writing—original draft; writing—review and editing; project administration.

## CONFLICT OF INTEREST STATEMENT

Jose Carlos Ferreira is on an advisory board and, since 2026, has been a consultant for Menarini Silicon Biosystems. Menarini is developing cEVT testing. Brynn Levy is on an advisory board for Menarini Silicon Biosystems. Peter Benn is a paid consultant to Natera, Inc., a provider of cell‐free DNA screening. He is on an advisory board for Menarini Silicon Biosystems.

## FUNDING INFORMATION

The authors received no specific funding for this work.

## ETHICS STATEMENT

The authors have nothing to report.

## Data Availability

Data sharing is not applicable to this article as no datasets were generated or analyzed during the current study.
